# The journey of medical field students: uncovering medical student syndrome, personality traits, and their interactions

**DOI:** 10.1186/s40359-025-02788-9

**Published:** 2025-05-09

**Authors:** Mohamed Abdelfatah Abdellatif, Heba A. Abdel Salam, Hossam Tharwat Ali, Salma Mohammed Hussein, Ahmed Ali Abdalla, Mohamed Khaled Khorkhash, Feras Ammar Alsabbagh, Nesreen Kamel Elsayed, Fatma Ahmed Ibrahim, Samar A. Amer

**Affiliations:** 1 Faculty of Medicine, Zagazig University, Zagazig, Egypt; 2 Department of Psychiatry, Faculty of Medicine, Zagazig University, Zagazig, Egypt; 3 Qena Faculty of Medicine, South Valley University, Qena, 83621 Egypt; 4 Department of Public health and community medicine department, Faculty of Medicine, Zagazig University, Zagazig, Egypt

**Keywords:** Medical student syndrome, Personality, Diagnostic and statistical manual of mental disorders, 5th edition, Anxiety-related illness, Medical field students, Hypochondriasis

## Abstract

**Background:**

Medical students commonly experience Medical Student Syndrome (MSS), a condition where they compare their vague symptoms to the medical problems and life-threatening diseases they are learning about in medical school, even though their health profile is free. Our research aims to investigate the symptoms of MSS (anxiety-related illness) and hypochondriasis, as well as their impact on the lives of students. Additionally, we aim to study various types of personalities and, finally, investigate the demographic determinants of MSS and their interactions with various personality types among medical students in Egypt in the period between September and December 2023.

**Methods:**

This analytical cross-sectional study targeted 300 students recruited from the medical field at Zagazig University. The data was collected using a self-administered questionnaire, which consisted of four main components: demographic data, the MSS questionnaire, the personality-type questionnaire, and the MSS’s impact. The collected data was coded and analyzed using R statistical software.

**Results:**

Out of the 300 medical field students recruited, 261 (87.0%) were Egyptian, 164 (54.67%) were female, and 216 (72.0%) were medical students without co-morbidity. 181 (60.33%) were aware of the MSS. Only 11 (3.67%) individuals met all criteria of the DSM-V for anxiety disorder, while 20 (6.67%) individuals met all criteria of the DSM-IV for hypochondriasis. The most common anxiety symptoms were difficulty sleeping (50.00%), lack of productivity or difficulty concentrating (44.0%), and rapid heart rate (31.67%). In terms of personality, being sympathetic and warm received the highest median of 6.00 while being critical and quarrelsome received the lowest median score of 2.00. The health anxiety score significantly negatively correlates with dependable and self-disciplined personality traits, while it significantly positively correlates with anxiety and upset personality traits.

**Conclusion:**

Around one-third of the sample experienced preoccupation with fears of having a serious disease, with a trivial number of students meeting the criteria for anxiety disorder or hypochondriasis. More than one-third reported negative impacts on sleep, productivity, concentration, and heart rate. Females, Egyptian students, those from rural areas, and dentistry students had higher anxiety scores, whereas medical students had the lowest scores.

**Supplementary Information:**

The online version contains supplementary material available at 10.1186/s40359-025-02788-9.

## Introduction

Medical field students commonly experience Medical Student Syndrome (MSS), a condition where they compare their vague symptoms to the medical problems and life-threatening diseases they are learning about in medical school, even though their health profile is free. The scientific community identified MSS using psychosomatic terms: “nosophobia” refers to an uncontrollable fear of a specific disease, while “hypochondria” refers to a persistent fear of a serious condition, resulting from delusions of contracting the disease and exaggerated minor symptoms despite appropriate medical evaluation [1].

MSS, as an anxiety disorder, has many negative impacts on the mental and physical health of medical students and may affect their academic and performance achievements. For example, they might feel patronized after seeking medical help for their symptoms, causing them to hesitate to seek medical help whenever the symptoms arise again. Even neglecting patients due to “illusion” symptoms undermines the patient-doctor relationship [2]. In addition to their health concerns and fear of physicians, both medical and non-medical students often self-diagnose and treat themselves based on their limited medical knowledge [3]. Surprisingly, in a study in 1966, few students reported positive effects, such as the facilitation of comprehending and absorbing information related to the symptoms they experienced [4].

Addiction, having family members in medical fields, having a history of mental illness, and the type of sex are all factors that can affect MSS. Neuroticism had a secondary impact on MSS’s development as a personality trait [5]. On the other hand, conscientiousness may result in higher stress levels [6]. Furthermore, some people’s alexithymic personality traits may influence their stress response, leading to symptoms not explained by medicine and a fear of illness [7, 8, 9]. However, we employed personality, a construct that encompasses five primary reflections derived from three tools (behavior, emotion, and cognition): conscientiousness, extraversion, openness, neuroticism, and agreeable [5, 6, 8, 9, 10, 11, 12]. Personality traits certainly affect the predisposition of individuals to certain psychiatric illnesses. Thus, it is important to consider different personality traits and characteristics when describing and studying the MSS [5, 6, 8].

Despite the 1960s description of the alleged MSS, its theoretical basis remains inadequate. Furthermore, contradictory studies conducted in the United Kingdom, Poland, Katowice, and Egypt found no significant difference in the levels of hypochondria and nosophobia among medical students compared to other students [13, 14, 15]. Therefore, it is crucial to explore and study the symptoms of MSS in order to increase awareness and develop effective management strategies for this syndrome among students. Previous and recent studies showed wide variations in prevalence rates in different geographical regions using different assessment tools [7,9,10, 13–15]. We conducted this study to enhance the general health condition of university students and lessen the effects of medical student syndrome, an anxiety-related illness, in Egypt by tracking the frequency of MSS. Our research aims to investigate the symptoms of MSS (anxiety-related illness) and hypochondriasis, as well as their attitude or impact on their lives; to study various types of personalities; and, finally, to investigate the demographic determinants of MSS and their interactions with various personality types among medical field students at Zagazig University, Egypt, in the period between September and December 2023.

.

## Subjects and methods

### Study design, setting, and participants

This cross-sectional survey design involved a total of 300 participants from Zagazig University in Egypt. The inclusion criteria required university undergraduate medical field students, including those studying medicine, veterinary medicine, dentistry, and pharmacy, to give their consent and complete the entire questionnaire. We excluded students with complex chronic illnesses or mental or psychological health issues that hindered their participation. The study adhered to the Strengthening the Reporting of Observational Studies in Epidemiology (STROBE) Checklist in its entirety.

### Sampling

#### Sample size

We determined the sample size using the formula: n = Z^2^ P (1 P)/d^2^, where n stands for sample size, Z for confidence level, P for expected prevalence, and d for precision or effect size. Examples, n.d.). We calculated it with a 95% confidence level, considering that 80% of the total study population were university students. Based on the wide range of MSS prevalence rates found in published studies (10–70–80%) [15], we will choose 50% because it gives us the largest sample size (324), which is what we need. We estimated that the smallest sample size was 300.

#### Sampling techniques

Using a multistage sampling technique, we collected a representative sample over the following stages: We selected a simple random sample from randomly selected classes, utilizing official websites and classroom social media channels such as Facebook, Twitter, official emails, and WhatsApp groups. In order to increase the sample size and boost the response rate, we sent follow-up messages and reminders to administrators and targeted demographics.

### Data collection

#### Preparation and validation of data collection tool

The data collection tool was prepared initially in English based on previous studies [7,9,10, 13–15]. Two bilingual healthcare professionals and one qualified medical translator initially translated it into Arabic. Two English-speaking translators performed back translation, after which they consulted the original panel to resolve any issues.

Two family medicine and two public health and community medicine experts from Zagazig University, Egypt, assessed the content validity, clarity, comprehension, and relevance of the questionnaire. We adjusted the questionnaire to ensure both relevance and feasibility among our population according to the experts’ comments. We then conducted a pilot study with 30 medical students.

The final questionnaire consisted of four main sections. The first section included sociodemographic data and factors related to health. The second part consisted of the MSS questionnaire, which met al.l the criteria of DSM-IV and DSM-V [7, 13, 14, 15]. Thirdly, the questionnaire included questions that implied personality types [9, 10], and the final section focused on the impact of the MSS. The details of the questionnaire are shown in Tables 1, 2, 3, 4 and 5.

**Table 1:** Demographic characteristics of the participants and their relationship with the illness anxiety score

Variable	Frequency	Illness anxiety score	*P*-value #
	(%) (N = 300)	Median (IQR)	
**Age** (years) **Median (IQR)**	22.00 (22.00 to 23.00)	*Spearman’s correlation* **= -0.12**	**0.04**
**Sex**			**< 0.01**
Female	164 (54.67)	6.00 (3.00 to 8.00)	
Male	136 (45.33)	4.00 (2.00 to 6.00)	
**Nationality**:			**0.02**
Egyptian	261 (87.00)	5.00 (3.00 to 7.00)	
Non-Egyptian	39 (13.00)	4.00 (1.50 to 5.50)	
**Residence**			**0.04**
Urban (with the family)	134 (44.67)	5.00 (2.25 to 7.00)	
Rural	102 (34.00)	5.00 (3.00 to 7.75)	
Urban (university hall / without the family)	64 (21.33)	4.00 (2.00 to 6.00)	
**Financial state**			0.18
More than sufficient income (Excellent / very good economic status)	224 (74.67)	5.00 (3.00 to 7.00)	
Insufficient income (Bad economic status)	60 (20.00)	4.00 (2.00 to 6.25)	
	16 (5.33)	5.50 (3.75 to 7.00)	
**Suffering from chronic diseases**			**< 0.01**
No	224 (74.67)	4.00 (2.00 to 7.00)	
Organic disease only	52 (17.33)	5.00 (3.00 to 8.00)	
Both organic and psychiatric diseases	13 (4.33)	7.00 (6.00 to 9.00)	
Psychiatric disease only	11 (3.67)	8.00 (5.50 to 8.00)	
**Family history of mental illness or disorder**	62 (20.67)	5.00 (3.00 to 7.00)	0.29
**Medical specialty**			**< 0.01**
Medicine	216 (72.00)	4.00 (2.00 to 6.00)	
Pharmacy	48 (16.00)	6.00 (3.75 to 8.00)	
Veterinary Medicine	28 (9.33)	7.00 (4.00 to 8.00)	
Dentistry	8 (2.67)	7.50 (5.50 to 8.50)	
**Academic year**	247 (82.33)	4.00 (2.00 to 7.00)	0.87
Third year or above	28 (9.33)	4.00 (1.00 to 7.00)	
Intern	13 (4.33)	5.50 (1.75 to 8.00)	
First or second year	12 (4.00)	5.00 (3.00 to 7.00)	
Postgraduate			
**Average academic performance**			
Very good to excellent (A-B)	251 (83.67)	5.00 (3.00 to 7.00)	0.07
Good (C)	36 (12.00)	5.50 (2.00 to 7.00)	
Poor (D)	11 (3.67)	8.00 (5.50 to 8.00)	
Fail	2 (0.67)	7.50 (7.25 to 7.75)	

**#** ***P*** **-value using Mann-Whitney U and Kruskal Wallis; bold means significance level at ≤ 0.05.**

**Table 2:** Illness anxiety items among the medical field students

Variable	Frequency (%) (N = 300)
**Do you know what medical syndrome is? #**	
No	119 (39.67)
Yes	181 (60.33)
**1. Do you experience preoccupation with fears of having or the idea that one has a serious disease based on the personal misinterpretation of bodily symptoms? †, ††**	
No	207 (69.00)
Yes	93 (31.00)
**2. The preoccupation persists despite appropriate medical evaluation and reassurance. ††**	
No	206 (68.67)
Yes	94 (31.33)
**3. There is a high level of anxiety about health and the individual is easily alarmed about personal health status. †**	
No	123 (41.00)
Yes	177 (59.00)
**4. Has this preoccupation lasted for at least 6 months? ††**	
No	227 (75.67)
Yes	73 (24.33)
**5. Illness preoccupation has been present for at least 6 months but the specific illness that is feared may change over that period. †**	
No	234 (78.00)
Yes	66 (22.00)
**6. Somatic symptoms are not present or if present are only mild in intensity. If another medical condition is present or there is a high risk for developing a medical condition (e.g. strong family history is present), the preoccupation is clearly excessive or disproportionate. †**	
No	229 (76.33)
Yes	71 (23.67)
**7. Have your bodily symptoms stopped you from working during the past six months or so? ††**	
No	227 (75.67)
Yes	73 (24.33)
**8. Have your bodily symptoms made you escape from working during the past six months or so? ††**	
No	215 (71.67)
Yes	85 (28.33)
**9. Do your bodily symptoms stop you from concentrating on what you are doing? ††**	
No	99 (33.00)
Yes	201 (67.00)
**10. Do your bodily symptoms stop you from enjoying yourself? ††**	
No	113 (37.67)
Yes	187 (62.33)
**11. When you experience unpleasant feelings in your body do you ever worry that they may be caused by a serious illness?**	
No	117 (39.00)
Yes	183 (61.00)
**12. Do you perform excessive health-related behaviors or exhibit maladaptive avoidance? †**	
No	135 (45.00)
Yes	165 (55.00)
**Total score ## Median (IQR)**	5.0 (3.0 to 7.0)
**Illness Anxiety Disorder Criteria (DSM-5)**	11 (3.67)
Fitting all criteria	289 (96.33)
No	
**Diagnostic and Statistical Manual of Mental Disorders, 4th Edition (DSM-4) Hypochondriasis Criteria**	
• Fitting all criteria	20 (6.67)
• No	280 (93.33)

**#** This question is for assessing the awareness of medical student syndrome and is not included in the total score. **/** Diagnostic and Statistical Manual of Mental Disorders, 5th Edition (DSM-5)

**##** Total score, from 0 to 12, is calculated for the 12 questions.

**†** Questions included in DSM-5 criteria along with having no psychiatric illness.

**††** Questions included in DSM-4 criteria along with having no psychiatric illness. Only one Yes answer to one question from questions number (7–10) is required for functional impairment item

**Table 3:** Experience of the medical field students with illness anxiety

Variable	Frequency (%) (N = 300)
**If you had it, what kind of illness did you initially self-diagnosed?**	
• No	189 (63.00)
• Organic disease	70 (23.33)
• Psychiatric disorder	28 (9.33)
• Both organic and psychiatric disorders	13 (4.33)
**Have you ever diagnosed yourself correctly?**	
• Yes	193 (64.33)
• No	107 (35.67)
**You think you are more prone to experiencing the symptoms of…...**	
• Both organic and psychiatric disorders	133 (44.33)
• Organic disease	88 (29.33)
• Psychiatric disorder	79 (26.33)
**Have you ever experienced this before becoming a student in the medical field?**	
• No	209 (69.67)
• Yes	91 (30.33)
**How do you react?**	
• None	135 (45.00)
• Care-avoidant type	76 (25.33)
• Care-seeking type	45 (15.00)
• Both	44 (14.67)
**Do you think this feeling affects you?**	
• No	170 (56.67)
• Negative effect	105 (35.00)
• Positive effect	25 (8.33)
**Have you ever diagnosed yourself using the internet before becoming a medical student?**	
• No	196 (65.33)
• Yes	104 (34.67)
**Which of the following best describes you?** ***(Multiple answers are allowed)***	
• Your fear and anxiety are disproportionate to the actual risk involved. For example, if someone is at high risk of developing a disease, they might not have nosophobia.	200 (66.67)
• The fear of getting sick interferes with other aspects of your daily life such as work school or relationships	85 (28.33)
• Your fear of illness is lasting not situational or temporary with anxiety persisting for six months or more	39 (13.00)

**Table 4:** Medical field students’ attitudes toward medical student syndrome

Variable	Frequency (%) (N = 300)
**What do you think about medical student syndrome?**	
• Phase in a medical student’s life	156 (52.00)
• Should be taken more seriously.	118 (39.33)
• Other	26 (8.67)
**Do you think that medical student syndrome plays a major role in the life of medical students?**	
• Agree	189 (63.00)
• Neutral	88 (29.33)
• Do not agree	23 (7.67)
**Do you think this feeling affects a person who suffers such syndrome?**	
• Negative effect	220 (73.33)
• No	62 (20.67)
• Positive effect	18 (6.00)
**Which of the following preventive measures do you think is most effective?**	
• Healthier lifestyle	209 (69.67)
• Sleep better.	139 (46.33)
• Talking to a professional	131 (43.67)
• Talking to a friend	73 (24.33)
• Others	22 (7.33)
**What is the name of the disease you thought you had contradicted? #**	
• Tumors	21 (7.00)
• Diabetes Mellitus	16 (5.33)
• Depression	15 (5.00)
• OCD	11 (3.67)
• Cardiac diseases	10 (3.33)
• General Anxiety disorders	6 (2.00)
• Psychiatric diseases (Not specified)	5 (1.67)
• Renal disease (Not specified)	4 (1.33)
• Asthma	3 (1.00)
• Rheumatoid arthritis	3 (1.00)
• ADHD	2 (0.67)
• Diabetes Insipidus	2 (0.67)
• IBD	2 (0.67)
• IBS	2 (0.67)
• Neurological disease (Not specified)	2 (0.67)
• PCOS	2 (0.67)
• Peptic ulcer	2 (0.67)
• Tremors	2 (0.67)
• Others **##**	24 (7.92)

**#** Multiple answers were allowed

**##** Others include Allergic Rhinitis, anemia, aneurysm, bipolar disorder, hypertension, hemorrhoids, multiple sclerosis, infarction, PTD, rheumatic fever, schizophrenia, PTSD, perforated drum, varicocele, and vitamin D deficiency

Abbreviations: OCD: Obsessive-compulsive disorder; ADHD: Attention deficit hyperactive disorder; IBD: Inflammatory bowel disease; IBS: Irritable bowel syndrome; PCOS: Polycystic ovary syndrome

**Table 5:** The personality types and their correlation coefficient illness anxiety score among medical field students

	Median (IQR)	Correlation Coefficient with the Illness anxiety score	*P*-value
Critical and quarrelsome	2.00 (1.00 to 4.00)	0.09	0.11
Dependable and self-disciplined	6.00 (4.00 to 6.00)	− 0.19	**< 0.01**
Anxious and easily upset	5.00 (3.00 to 6.00)	0.34	**< 0.01**
Open to new experiences and complex	4.50 (3.00 to 6.00)	0.01	0.86
Reserved and quiet	5.00 (4.00 to 6.00)	− 0.08	0.18
Sympathetic and warm	6.00 (5.00 to 7.00)	0.17	**< 0.01**
Conventional and uncreative	4.00 (2.00 to 5.00)	− 0.09	0.14
Disorganized and careless	3.00 (2.00 to 5.00)	0.08	0.16
Calm and emotionally stable	4.00 (3.00 to 5.00)	− 0.27	**< 0.01**

**Bold means significance level at ≤ 0.05.**

The data collection process took place in the period between September 1st and December 31st, 2023. For each logging email, we allowed only one answer to prevent duplicate responses. Participants provided their consent to participate before completing and submitting the survey.

### Data analysis

We organized the patients’ characteristics in a Microsoft Excel sheet, and then imported and analyzed them using R Statistical Software (Version 2023.12.1 + 402). We used frequencies and percentages to describe the categorical variables for baseline demographic characteristics. We assessed the normality of the continuous variables using the Shapiro-Wilk test. We used the median and interquartile range (IQR) to describe the non-parametric variables. We recorded the questions assessing health, illness, and anxiety as 1 for correct answers and 0 for incorrect ones, resulting in a total score ranging from 0 to 12. We described the answers as frequencies and percentages and displayed the total score as the median and the interquartile range (IQR). We described participants meeting all criteria of DSM-IV and DSM-V as frequency and percentage.

We used the median (IQR) for the total score of personality traits. The answers were recorded as follows: strongly disagree = 1, moderately disagree = 2, disagree a little = 3, neither agree nor disagree = 4, agree a little = 5, moderately agree = 6, and strongly agree = 7. We assessed the factors associated with health, illness, anxiety, and personality characteristics using the Mann-Whitney U and Kruskal-Wallis tests. Spearman’s correlation analysis was used to assess the correlation between age, health illness anxiety score, and scores of personality characteristics. A *p*-value of ≤ 0.05 was considered significant.

### Ethical considerations

The study was approved by the Institutional Review Board (or Ethics Committee) of the research center at Zagazig University (ZU-IRP#10971–25/7-2023) following the Declaration of Helsinki. Participant’s anonymity was preserved through the survey and all participants consented before filling in the survey.

## Results

### Students’ demographic characteristics

The final analysis included 300 students, with 261 students (87.00%) being Egyptian. The median and IQR of age were 22.00 (22.00 to 23.00) years. 164 individuals (54.67%) were female, while 216 (72.0%) were specializing in medicine. The majority of the students, 247 (82.33%), were in their third academic year or above, and 74.67% did not suffer from any chronic organic or psychiatric diseases. Table 1 displays the details of the demographic characteristics.

### Experience and attitudes of the students with health, illness, MSS, anxiety, and associated factors

Most of the students, 181 (60.33%), were aware of the medical student syndrome. Only 93 (31.00%) experienced preoccupation with fears of having or the idea that one has a serious disease based on the personal misinterpretation of bodily symptoms while 73 (24.33%) had preoccupation lasted for at least 6 months. In 94 participants (31.33%), the preoccupation persisted despite appropriate medical evaluation and reassurance. The participants had a median (IQR) score of 5.0 (3.0 to 7.0) for the health illness anxiety.

Only 11 (3.67%) individuals met all criteria of DSM-V for illness anxiety disorder while 20 (6.67%) individuals met all criteria of DSM-IV for hypochondriasis. Most participants, 200 (66.67%), thought fear and anxiety were disproportionate to the actual risk involved, while only 105 (35.00%) thought that feeling affected them negatively. The most involved diseases in illness anxiety among our participants were tumors (7.00%), followed by diabetes mellitus (5.33%), depression (5.00%), and obsessive-compulsive disorder (3.67%) (Table 1S). Tables 2 and 3 detail the participants’ experiences with health, illness, and anxiety.

**Fig. 1:**
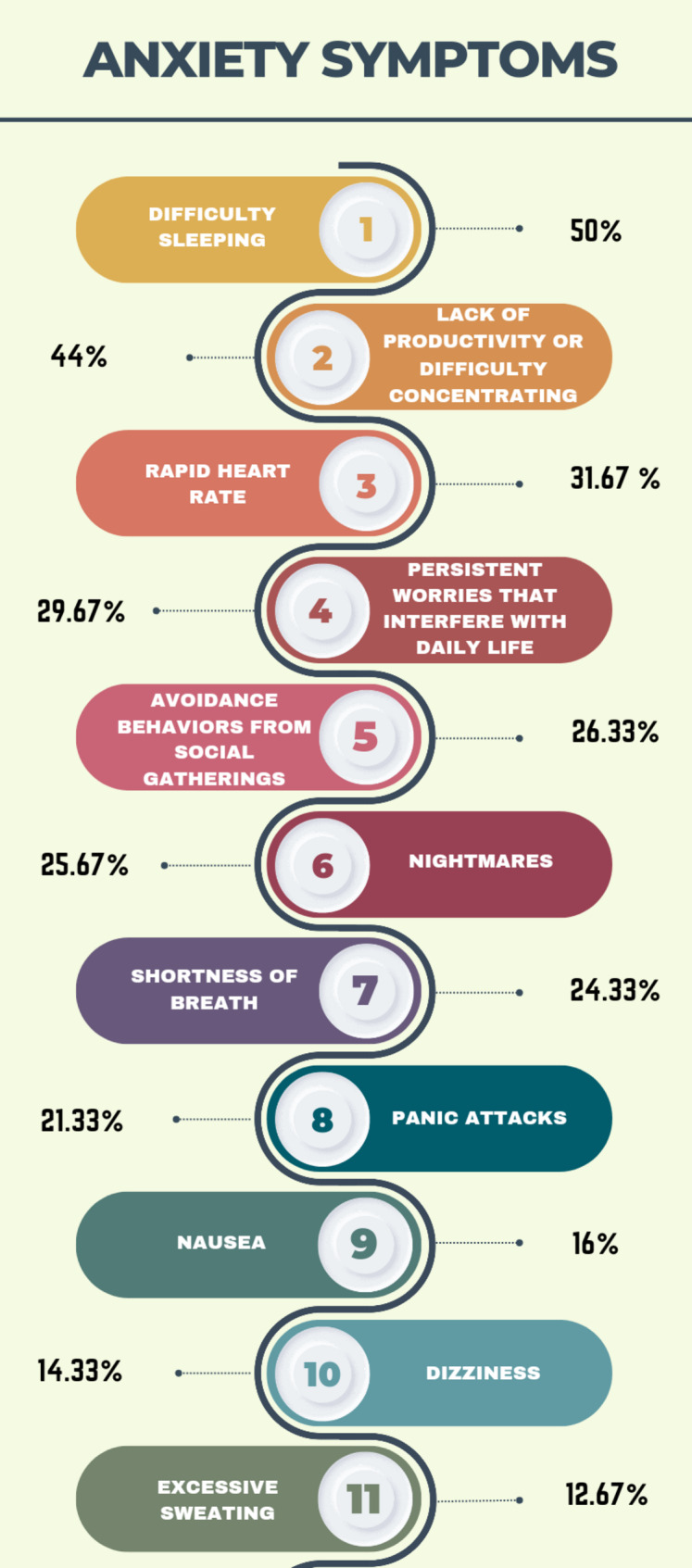
Percentages of anxiety symptoms among medical field students

***Of the anxiety symptoms***, the most common were difficulty sleeping (50.00%), lack of productivity or difficulty concentrating (44.00%), and rapid heart rate (31.67%) (Fig. 1***and*** Table 2S).

### Medical field students attitudes towards MSS, anxiety, and associated factors or determinants

The participants showed variable attitudes toward such conditions with 189 (63.00%) participants agreeing that medical student syndrome plays a major role in medical students’ lives. 209 individuals (69.67%) perceived a healthier lifestyle as the most effective preventive measure. Table 4 displays the details of the participants’ attitudes.

Regarding the factors associated with health illness anxiety, females were associated with higher scores (median of 6.00) compared to males (median of 4.00) (*p*-value < 0.01) (Table 1). Similarly, Egyptian participants had higher scores (median of 5.00 compared to 4.00; *p*-value = 0.02). Participants specializing in medicine had the lowest anxiety scores (median of 4.00), while those in dentistry had the highest anxiety scores (median of 7.50) (*p*-value < 0.01).

### Perceived personality characteristics among the medical field students

Being sympathetic and warm received the highest median (IQR) of 6.00 (5.00 to 7.00) while being dependable and self-disciplined received a median (IQR) of 6.00 (4.00 to 6.00). On the other hand, the lowest score was for being critical and quarrelsome, with a median (IQR) of 2.00 (1.00 to 4.00), followed by disorganized and careless, with a median (IQR) of 3.00 (2.00 to 5.00).

### The interrelations between the MSS, and the perceived personality characteristics

The Spearman’s correlation analysis revealed a weak negative correlation between age and health illness anxiety score (ρ = − 0.12, *p*-value = 0.04). Similarly, the health anxiety score was negatively correlated with being dependable and self-disciplined (ρ = − 0.19, *p*-value < 0.01). The personality characteristic of being anxious and easily upset was positively correlated with the health illness anxiety score (ρ = 0.34, *p*-value < 0.01). Table 5 displays the details of Spearman’s correlation analysis.

## Discussion

For many decades, there has been a persistent myth in medical schools about a condition known as Medical Students Syndrome, or Medical Students’ Disease, which affects young people completing medical degrees [15]. However, everyone agrees that the medical course is one of the most challenging, requiring students to focus their efforts, dedicate themselves to their studies, and face intense competition [16].

### Demographics of the study students

Our participants had a median (IQR) age of 22.00 (22.00 to 23.00) years with most of them (72.0%) specializing in medicine and nearly half of them are males. Previous studies had disparities regarding the participating students’ characteristics. Abdullah et al. [17]. included participants with a mean age of 20.6 years, ranging from 17 to 25 years. Females comprised most of the sample at 71% (*n* = 335), while the nationalities of the participants included Arabs, Emiratis, and non-Arabs. The sample consisted of foundation-year students, preclinical students, and clinical students [17]. Furthermore, Shehata and Abdeldaim [18] included 500 medical students in the Tanta Faculty of Medicine with a mean age of 20.36 years with 43.8% of them being males. More than half of the students (58.2%) lived in rural areas compared to only 34% in our study. Around 60% of the students did not have a positive family history of chronic diseases [18] while only 20.67% did in our study.

### Experience, and frequency of the medical field students with illness anxiety disorder

Regarding the frequency of MSS, among Egyptian medical field students who met the DSM-IV criteria for hypochondriasis, 20 (6.67%) had anxiety disorder. According to Abdullah et al.‘s (2023) study [17], 70.8% (334) of the study population had MSS; in Tanta Shehata and Abdeldaim’s (2022) research [18], 78.8% had hypochondriasis. However, in Santa Catarina, Brazil, in 2015, there was a prevalence of 35.5% and 32.8%, respectively, for anxiety symptoms and depression [19], and 66.8% for hypochondriasis, also known as MSS. In Vasconcelos et al., 2015 [20], anxiety prevalence was 19.7%, and depression prevalence was 5.6%. A 2017 study involving 700 medical students in economically developing countries like Egypt revealed a high prevalence of anxiety symptoms at 73% and depression at 65% [21]. In Turkey, they found a prevalence rate of 35.8% for symptoms of medium and moderate anxiety, 30.5% for medium and moderate depression, and 8.5% for severe depression [22].

The disparities in prevalence rates reported in the literature could have resulted from variations in the student body’s geographic and cultural backgrounds, as well as from the techniques and kinds of surveys employed to collect the data. The large number of common symptoms described in the literature could be due to several things, such as the use of different psychometric instruments (the Self-Reporting Questionnaire (SRQ-20), the Beck Anxiety Inventory (BAI), and the Beck Depression Inventory (BDI), each with a different level of sensitivity and specificity). Of all the social groups, college students are more likely to develop anxiety and depression disorders. and the fact that students from different countries have very different ways of expressing and recognizing their emotions, both in terms of how strongly they are felt and how they are reported among medical students on several continents [23, 24].

The present study found that most students, 181 (60.33%), were aware of the medical student syndrome. Abdullah et al. (2023) [17] found that, among the students surveyed, 55.9% had previously heard about MSS (*n* = 264). Only 93 (31.00%) experienced preoccupation with fears of having or the idea that one has a serious disease based on the personal misinterpretation of bodily symptoms while 73 (24.33%) had preoccupation lasted for at least 6 months. In 94 students (31.33%), the preoccupation persisted despite appropriate medical evaluation and reassurance. The students had a median (IQR) score of 5.0 (3.0 to 7.0) for the health illness anxiety.

Of the students, 200 (66.67%) believed that their level of fear and anxiety was not commensurate with the actual risk involved, whereas only 105 (35.00%) felt that their feelings had a negative impact on them. Many theories explain this by suggesting that medical field students, who learn a lot about potentially fatal diseases during their training, may look for symptoms that affect them and worry that they have a serious illness or are exaggerating minor ones. This may prompt them to self-diagnose a specific somatic disease, a condition known as hypochondria. This makes them more likely to experience anxiety or anxiety disorder, which is a mental illness. However, a lack of experience and knowledge could lead to inaccurate diagnoses. Exposure to the symptoms of patients that students interact with, exposure to a stressful and competitive work environment, and students’ emotional responses could all be contributing factors to the emergence of this phenomenon [17].

The present study found that the most common anxiety symptoms were difficulty sleeping (50.00%), lack of productivity or difficulty concentrating (44.0%), and rapid heart rate (31.67%). The participants showed variable attitudes toward such conditions with 189 (63.00%) participants agreeing that medical student syndrome plays a major role in medical students’ lives. 209 individuals (69.67%) perceived a healthier lifestyle as the most effective preventive measure.

### Differences in the illness anxiety score across the demographic variables

#### Age determinants

The current study’s results revealed a weakly negative correlation (*r* = -0.12, *p*-value = 0.04) between the health illness anxiety score and age. We found that students between the ages of 16 and 20 were 2.02 times less likely to have felt they had MSS compared to students in the age range of 21 to 25 (*p* = 0.001). In agreement with Abdullah et al. (2023) [17], this type of environment subjects young medical students to extreme stress. They are also more likely than their peers to experience anxiety, depression, and other mental health issues [18].

#### Sex as an MSS determinant

The current study revealed that females had higher illness anxiety scores (median of 6.00) than males (median of 4.00), with a *p*-value of less than 0.01. In agreement with the researcher, women were more likely than men to suffer from hypochondriasis [18]. Specifically, Backović et al. (2012) [25] Faravelli et al., (2013) [26]; Karger, (2014) [27], Moskalewicz et al., (2015) [28]; and Fawzy & Hamed (2017) [21] found that women are more likely than men to display morbid fear for their health among the 606 students from the Silesian region [29].

Studies hypothesize that there are several potential reasons for this gender dependence, including Neuroendocrine factors linked to hormonal cycles, as well as psychological and physiological factors, which may contribute to this gender dependence, given that women are more likely to claim specific illnesses. However, we must approach the diagnosis of anxiety disorder in this population with caution, considering the family history of somatic diseases and the patient’s past medical conditions. Compared to men, women respond faster to the first signs of disease, and they are more likely to focus on their bodies and any health issues they may be experiencing. Moreover, women are characterized by a greater need to talk about their health with others. They also more often decide to contact a specialist doctor, who can address their health worries. The stereotype of men presenting to health care professionals late and less often in our culture appears to hold [29]. In addition to environmental factors and social, work, and family stressors, females perform numerous social roles outside of the classroom, potentially contributing to their higher rates of burnout. These roles consume the energy and time required for learning and resting. Furthermore, female students tend to have a lower stress threshold, rather than experiencing more stressors than their male counterparts [30, 31].

#### Regarding the academic year

More than three-quarters of those with hypochondriasis (77.2%) were students whose parents did not work in the medical field. Furthermore, compared to their first- and fourth-year counterparts, sixth-year students (clinical year students, fourth to sixth medical year) have the highest prevalence across all burnout syndrome dimensions. This outcome is in line with the findings of Melaku et al. (2015) [32], who investigated and concluded that among 329 medical field students who move into more advanced stages of their education, their level of burnout rises. According to Santen et al. (2010) [33], among all the students in various years, there is not a statistically significant difference.

#### Regarding the academic year as an MSS determinant

More than three-quarters of hypochondriasis patients (77.2%) were students whose parents did not work in the medical field. Additionally, sixth-year students (clinical year students, fourth to sixth medical year) have the highest prevalence across all dimensions of burnout syndrome when compared to their first- and fourth-year counterparts. Melaku et al. (2015) [32] found that 329 medical students’ degree of burnout increases as they progress through higher educational stages, which is consistent with this result. Santen et al. (2010) [33] state that there is not a statistically significant difference between all of the students in different years.

A medical student’s academic path entails a demanding and drawn-out daily schedule of activities, which includes long journeys that interfere with socializing, unwinding, and even sleeping [34]. Bertani et al. (2020) [35] discovered that several academic factors could be responsible for this high level of stress and anxiety, including a heavy workload, the amount of time spent on hospital internships and curricular lessons, a competitive environment, difficult and remembered subjects, and frequent exams. Furthermore, intricate interactions with patients and their families may exacerbate the psychological and emotional strain of the degree course.

In addition, those from rural areas (55.6%, 51.5%, and 58.6%, respectively), and Egyptian students had higher scores (median of 5.00 compared to 4.00; *p*-value = 0.02) compared to other nationalities in agreement with Shehata and Abdeldaim, (2022) [18].

#### Comorbidities, and family history

Despite The majority of students (74.67%) did not have any long-term mental or organic health conditions. The most frequent conditions linked to illness anxiety among the participants in this study were tumors (7.00%), diabetes mellitus (5.33%), depression (5.00%), and obsessive-compulsive disorder (3.67%). 77.7% of medical students who were hypochondriacs had never taken sleep or psychiatric medication, and the majority of them (84.8%) had never seen a psychiatrist [18].

Contact with the patient’s anguish, pain, and even death events that also induce anxiety and stress in medical field students is another crucial component. Due to the students’ lack of time or the school’s negligence, it is difficult to take care of one’s health, which raises the risk of developing burnout syndrome and experiencing symptoms of anxiety and depression [36].

More than half of those with hypochondriasis (58.4%) did not have a family history of chronic illnesses. According to Szczurek et al. (2021) [13], students who received treatment for other mental disorders or had a family history of mental disorders indicated on the questionnaire were much more likely to report having hypochondria and a fear of getting sick. This could be the result of specific disorders coexisting [37].

#### Comparing medicine to dentistry

Participants specializing in medicine had the lowest anxiety scores (median of 4.00), while those in dentistry had the highest anxiety scores (median of 7.50) (*p*-value < 0.01). The environment places a great deal of stress on young medical students. Compared to their peers, they also have a higher likelihood of experiencing anxiety, depression, and other mental health issues. However, it is noteworthy to remember that some students’ well-being is not, and may never be, in danger [18].

### Association between MSS and other mental (psychiatric) illnesses

Our results ensure that monitoring of anxiety and depression levels among medical students is important. Consistently, a multi-systemic and comprehensive approach with specific policies, should address increased levels of anxiety and depression. Examples of this approach include the VMS Wellness Program of the Vanderbilt School of Medicine and the Dutch 4T-CABS (Four-Tier Continuum of Academic and Behavioral Support). The integration of such programs in the university curriculum could potentially improve the psychophysical well-being of students, reduce burnout, and improve their performance [38].

#### Personality is a strong predictor of well-being

We detected the highest score for being sympathetic and warm, followed by dependable and self-disciplined. Conversly, the lowest score was for being critical and quarrelsome, followed by disorganized and careless. However, the effects of personality on students’ well-being are complex, integrating a combination of multiple traits and the situation. Well-being can be defined differently by everyone out of each person’s unique experiences and interests, however, all the components of health and well-being (i.e., physical, emotional, social, cognitive, and spiritual) are interdependent and influenced by personality [39].

We revealed a negative correlation between health anxiety illness and being dependable and self-disciplined (*r* = − 0.19, *p*-value < 0.01). On the other hand, the personality characteristic of being anxious and easily upset was positively correlated with the health anxiety score (*r* = 0.34, *p*-value < 0.01) which is in line with the literature. This association includes two personality dimensions belonging to the area of internalization, marked by depression, anxiety, anhedonia, and social withdrawal [27]. Our findings confirm the relationship between personality and anxiety symptoms and suggest the importance of early identification of maladaptive personality traits. Nevertheless, since the study is transversal, it does not allow the demonstration of a unilateral causal relationship; it could also prove how the onset of anxiety or mood disorders can lead to the development of maladaptive behavioral characteristics.

### The impact of MSS on the student’s life

Although only a minority is fitting the criteria for illness anxiety disorder (3.67%) and hypochondriasis (6.67%), more than half of the participants (55%) react to the illness anxiety symptoms with 25.33% exhibiting care-avoidant type. Shehata and Abdeldaim [18] reported that more than half of the students with hypochondriasis (53.6%) experienced mild stimuli as they ignored them. In response to these stimuli, 33% of the students investigated further. These feelings lasted for days for 24.9% of students. On the other hand, Sadiq et al. [40] found that 13.9% of their medical students visited a psychiatrist, and 22.8% reported taking psychiatric medications before. Interestingly, nearly a third of our participants (30.33%) admitted experiencing such symptoms before the medical school. However, other studies on Saudi Arabian medical students revealed that 40.9% of them admitted to self-medicating for their symptoms [3]. Abdullah et al. [17]. reported that they asked participants who felt they had a disease they studied to elaborate on their feelings. Among all participants, 28% sought medical assistance (*n* = 92), and 37% mentioned a presumed disease diagnosis (*n* = 34).

While half of the study participants believed that MSS is a phase in the student’s life, most of them agree it has a negative effect and play a major role in their lives. The results of our study may have some implications for medical students’ health policy. The high frequency of mental health problems among medical filed students should raise the institutions’ and academicians’ attention. This is why some authors analyzing this problem postulate that mental health services should be available, accessible, and affordable at universities for every students on a regular basis [41, 42].

### Strengths, and limitations

This study had many strengths, including being the first to highlight the interactions between medical students’ syndromes and personality types. Moreover, we analyze multiple determinants of MMS and its impact, paving the way for further research. Although we used three validated tools in this study, including the MSS questionnaire, which met al.l the criteria of DSM-IV and DSM-V, and the personality types questionnaire among medical field students (7, 9–10, 13, 14, 15), the condition still requires further clinical diagnosis. Moreover, the self-administered nature of the questionnaire ensures voluntary and transparent participation. This approach mitigates the potential influence of wish-related bias—the perception of mental health issues as social stigma—but is associated with all the drawbacks of cross-sectional studies, including self-recall biases in the results. Despite the potential impact of a single-setting study on generalizability, its relatively large size enhances its power. Finally, the study does not elaborate on the implications of MSS on the medical profession and patient care, despite briefly discussing the attitudes of medical field students regarding MSS and finding no correlation with their academic performance.

## Recommendations

Given the aforementioned statistics, we recommend conducting further research to delve deeper into the implications of MSS on academic performance, the medical profession, and patient care. Additionally, we recommend collaborating with psychiatry to develop specific evidence-based programs aimed at reducing the impact of MSS and anxiety-related illness among medical field students. These programs range from individually targeted interventions like relaxation techniques, meditation, coping strategies, and mindfulness to structural interventions like workload redistribution, increased practical activities, and improved dialogue between students and teachers.

Medical field undergraduate courses should integrate these approaches with the traditional course to reduce the impact of MSS and anxiety-related illness, while frontal lectures adopt a transversal teaching model akin to those in business schools. This model is based on problem-solving, stimulating creative thinking, and developing personal projects (42–43).

Mental health wellbeing clinics should be available for free and provide anonymous and remote primary mental care services to all medical field students for early diagnosis and effective management.

Furthermore, we recommend conducting similar studies on a national and international level for better exploration of the MMS and to provide region- and international-specific insights that are valuable for tailoring interventions.

## Conclusions

According to our study findings, around one-third of the sample experienced preoccupation with fears of having a serious disease based on personal misinterpretations. A trivial number of students met all of DSM-V’s criteria for anxiety disorders and all of DSM-IV’s criteria for hypochondriasis. Further large multicenter studies based on clinical diagnoses are recommended to explore the MSS and provide generalizable results.

## Electronic Supplementary Material

Below is the link to the electronic supplementary material.

Supplementary Material 1

## Data Availability

The data generated in this study are presented in the manuscript and supplementary data. Any further reasonable requests should be directed to the corresponding author.

## Abbreviations

BAI: Beck Anxiety Inventory
BDI: Beck Depression Inventory
DSM-4: Diagnostic and Statistical Manual of Mental Disorders, 4th Edition
DSM-5: Diagnostic and Statistical Manual of Mental Disorders, 5th Edition
FPS: Faculdade Pernambucana de Sade
IQR: Interquartile range
MSS: Medical Student Syndrome
STROBE: Strengthening observational studies in epidemiology reporting
SRQ-20: The Self-Reporting Questionnaire
