# Supplementary material for: The journey of medical field students: uncovering medical student syndrome, personality traits, and their interactions
Source: BMC Psychol. 2025 May 9;13:490. doi: 10.1186/s40359-025-02788-9 (PMC12065215; doi:10.1186/s40359-025-02788-9)
Supplement: Supplementary file 1 — Supplementary Material 1 [file 40359_2025_2788_MOESM1_ESM.docx]

**13- SUPPLEMENTARY TABLES**

| **Table 1S: Names of the involved diseases in the illness anxiety among medical field students** | |
| --- | --- |
| **What is the name of the particular disease you thought you had contradicted? #** | **Frequency (%) (N=300)** |
| - Tumors | 21 (7.00) |
| - Diabetes Mellitus | 16 (5.33) |
| - Depression | 15 (5.00) |
| - OCD | 11 (3.67) |
| - Cardiac diseases | 10 (3.33) |
| - General Anxiety disorders | 6 (2.00) |
| - Psychiatric diseases (Not specified) | 5 (1.67) |
| - Renal disease (Not specified) | 4 (1.33) |
| - Asthma | 3 (1.00) |
| - Rheumatoid arthritis | 3 (1.00) |
| - ADHD | 2 (0.67) |
| - Diabetes Insipidus | 2 (0.67) |
| - IBD | 2 (0.67) |
| - IBS | 2 (0.67) |
| - Neurological disease (Not specified) | 2 (0.67) |
| - PCOS | 2 (0.67) |
| - Peptic ulcer | 2 (0.67) |
| - Tremors | 2 (0.67) |
| - Others **##** | 24 (7.92) |
| **#** Multiple answers were allowed  **##** Others include Allergic Rhinitis, anemia, aneurysm, bipolar disorder, hypertension, hemorrhoids, multiple sclerosis, infarction, PTD, rheumatic fever, schizophrenia, PTSD, perforated drum, varicocele, and vitamin D deficiency.  Abbreviations: OCD: Obsessive compulsive disorder; ADHD: Attention deficit hyperactive disorder; IBD: Inflammatory bowel disease; IBS: Irritable bowel syndrome; PCOS: Polycystic ovary syndrome | |

| **Table 2S: Anxiety symptoms among medical field students** | |
| --- | --- |
| **Do you suffer from any of the following symptom of anxiety about health? #** | **Frequency (%) (N=300)** |
| - Difficulty sleeping | 150 (50.00) |
| - Lack of productivity or difficulty concentrating | 132 (44.00) |
| - Rapid heart rate | 95 (31.67) |
| - Persistent worries that interfere with daily life | 89 (29.67) |
| - Avoidance behaviors from social gathering | 79 (26.33) |
| - Nightmares | 77 (25.67) |
| - Shortness of breath | 73 (24.33) |
| - Panic attacks | 64 (21.33) |
| - Nausea | 48 (16.00) |
| - Dizziness | 43 (14.33) |
| - Excessive sweating | 38 (12.67) |
| **#** Multiple answers were allowed | |
